# Early Freezing of Gait: Atypical versus Typical Parkinson Disorders

**DOI:** 10.1155/2015/951645

**Published:** 2015-02-15

**Authors:** Abraham Lieberman, Aman Deep, Rohit Dhall, An Tran, Ming-Jai Liu

**Affiliations:** Muhammad Ali Parkinson Center (MAPC), Barrow Neurological Institute, St. Joseph's Hospital and Medical Center, Phoenix, AZ, USA

## Abstract

In 18 months, 850 patients were referred to Muhammad Ali Parkinson Center (MAPC). Among them, 810 patients had typical Parkinson disease (PD) and 212 had PD for ≤5 years. Among the 212 patients with early PD, 27 (12.7%) had freezing of gait (FOG). Forty of the 850 had atypical parkinsonism. Among these 40 patients, all of whom had symptoms for ≤5 years, 12 (30.0%) had FOG. FOG improved with levodopa in 21/27 patients with typical PD but did not improve in the 12 patients with atypical parkinsonism. FOG was associated with falls in both groups of patients. We believe that FOG unresponsive to levodopa in typical PD resembles FOG in atypical parkinsonism. We thus compared the 6 typical PD patients with FOG unresponsive to levodopa plus the 12 patients with atypical parkinsonism with the 21 patients with typical PD responsive to levodopa. We compared them by tests of locomotion and postural stability. Among the patients with FOG unresponsive to levodopa, postural stability was more impaired than locomotion. This finding leads us to believe that, in these patients, postural stability, not locomotion, is the principal problem underlying FOG.

## 1. Introduction

Freezing of gait (FOG) is characterized by an inability to walk in which the patient's feet appear to be “stuck,” while the upper body continues to move. The forward progression of the feet is markedly reduced despite the intention to walk [1]. FOG is usually precipitated by turning, by walking through a doorway, by walking in a confined space, and by dual tasking [1, 2]. Turning, walking through a doorway, and walking in a confined space have in common rapid and frequent shortening of step length [3, 4]. Because FOG is characterized by a shortening step length, it is usually thought of as a disorder of locomotion. But FOG is also characterized by falls, suggesting it is also a disorder of postural stability [1]. To study the influence of locomotion and postural stability on FOG, we compared FOG in patients with typical Parkinson disease (PD) and atypical parkinsonism, disorders characterized by different degrees of impaired locomotion and postural stability. Although the etiology, pathology, and pathophysiology of these disorders are different, the phenomenon of FOG appears similar.

FOG occurs in Parkinson's disease (PD). Its prevalence has been variously reported, depending on referral patterns and the mix of early and late disease patients. In a longitudinal analysis of 800 patients with early, untreated PD, part of the DATATOP study, 57 of 800 patients (7.1%) had FOG on entry, and 193 patients (26%) had FOG upon completion of the study [5]. In a cross-sectional analysis of a different series by the same author, 318 of 990 patients (32%) had FOG [6]. FOG is more common late in PD, with as many as 91 of 172 patients (53%) demonstrating it [7]. FOG in PD sometimes responds to levodopa and sometimes does not [5–7]. The events preceding and during a freeze are characterized by a decrease in step length, a decrease in range of movement of the hips, knees, and ankles, and an increase in falls [1, 8–11]. In a study of 401 PD patients seen in one year, 44 patients (11%) fell repeatedly. Twenty-one of these patients (48%) had FOG [8].

FOG occurs in atypical parkinsonism disorders including multiple-system atrophy (MSA), progressive supranuclear palsy (PSP), Lewy body dementia (LBD), and normal pressure hydrocephalus [1, 12]. FOG also occurs in progressive primary freezing of gait (PPFG), a disorder characterized by freezing without evidence of PD [13, 14]. It is unclear if PPFG is a separate disorder or is part of the spectrum of disorder of PSP [13, 14]. PPFG does not respond to levodopa.

Atypical parkinsonism progresses more rapidly than PD, and most patients with atypical parkinsonism, unlike patients with PD, are disabled in 10 years or less. To compare FOG in atypical parkinsonism and typical PD, we chose those patients who had been diagnosed with atypical parkinsonism or typical PD for five years or less.

## 2. Materials and Methods

In an 18-month period, January 1, 2013, to June 30, 2014, we saw 850 new patients, who were referred with a diagnosis of PD or atypical parkinsonism. Of these, 212 patients (25%) had PD ≤ 5 years, and 27 of the 212 had FOG. Thus 13% of the 212 patients with early PD had FOG. All 27 patients improved on levodopa. Ten of the 27 patients (37%) had dyskinesia. Of the 27 patients, FOG improved on levodopa treatment in 21 (78%). In six patients, FOG did not improve on levodopa. Fifteen patients (56%) fell repeatedly.

Among the 850 patients, 40 patients (4.7%) were diagnosed as having atypical parkinsonism. All 40 had their disorder for ≤ five years. Two patients were diagnosed with LBD, three with PPFG, three with MSA, and four with PSP. Twelve of the 40 patients with atypical parkinsonism had FOG. All 12 patients fell repeatedly. These 12 atypical parkinsonism patients had at one time been on levodopa for at least four months. Two MSA patients had briefly responded to levodopa. At the time of the analysis, none of the 12 atypical patients were on levodopa.

Accepted criteria were used to diagnose typical PD [15, 16] and atypical parkinsonism [17–19]. Patients with typical PD were assessed using the motor portion of the MDS-UPDRS [20, 21]. FOG was assessed using the five-point (from 0 to 4) FOG subtest of the UPDRS. A score of ≥2 was considered abnormal. Locomotion was assessed using the five-point gait subtest of the UPDRS. A score of ≥2 was considered abnormal. Locomotion was also assessed by having the patient walk 7.67 meters (25 feet) and counting the number of steps the patient took [9]. Postural stability was assessed using the five-point “pull subtest” of the UPDRS. A score of ≥3 was considered abnormal. Postural stability was also assessed by asking the patient to stand on one leg. An inability to stand on either leg for <3 seconds was considered abnormal [22]. Patients were also compared using the six-point (from 0 to 5) Hoehn and Yahr scale.

All 27 typical PD patients were on levodopa and all had responded as determined by a 30% or greater decrease in the motor portion of the MDS-UPDRS. All 27 patients had been evaluated in an “off” state 16 hours after their last levodopa dose. All had FOG in their “off” state. All were then given a test dose of levodopa up to 200% of their usual dose. They were then evaluated in their “on” state. Among the 27 patients, FOG improved in 21 (78%). FOG did not improve in 6 patients (22%). We studied three groups of patients with FOG:

1. a group of 12 atypical parkinsonism patients whose FOG did not respond to levodopa: these patients fell repeatedly (>2 falls per month);
2. a group of 6 typical PD patients whose FOG did not respond to levodopa: these patients fell repeatedly (≥2 falls per month);
3. a group of 21 PD patients whose FOG did respond to levodopa: these patients fell less often (≤1 fall per month).

Although there are anatomical, pathological, and structural differences between the atypical parkinsonism and the typical PD patients, we grouped the 6 typical PD patients whose FOG did not respond to levodopa with the 12 atypical parkinsonism patients whose FOG did not respond to levodopa, consolidating the three groups into two. We then compared these 18 patients to the 21 typical PD patients whose FOG did respond to levodopa (Tables 1 and 2).

We compared age, disease duration, falls, and step length between both groups using a 2-tailed* t*-test at the 0.05 level of significance. We compared the five-point Hoehn and Yahr staging system, the five-point FOG subtest, the five-point “pull subtest,” the five-point gait subtest, and the ability or inability to stand on one foot for < 3 seconds with odds ratios using MedCalc, version 14.17.0 (MedCalc Software, Ostend, Belgium).

All patients were informed that their evaluations might be used in scientific publications but that they, personally, could not be identified. The retrospective study was approved by the Institutional Review Board of St. Joseph's Hospital and Medical Center in Phoenix, Arizona, USA.

## 3. Results

In Table 1, age, disease duration, and Hoehn and Yahr stage are compared between the 18 patients whose FOG did not respond to levodopa and the 21 patients whose FOG did respond to levodopa. Disease duration was significantly shorter in the 18 patients whose FOG did not respond to levodopa. Clinically, the importance of the observation is moot, because we chose only patients who had their disease for ≤ five years. Hoehn and Yahr stage was significantly worse in the group of 18 patients whose FOG did not respond to levodopa, and falls were more frequent.

In Table 2, FOG, the “pull subtest,” and the ability to stand on one foot were significantly worse in the group of 18 patients whose FOG did not respond to levodopa. Step length was significantly decreased (worse) in this group. The gait subtests were similar. Tests of postural stability, the “pull subtest,” and the ability to stand on one foot were more impaired than tests of locomotion, that is, the gait subtest and step length.

## 4. Discussion

Patients with atypical parkinsonism and patients with typical PD whose FOG did not respond to levodopa have a more “debilitating” disorder than patients with typical PD whose FOG did respond to levodopa. The patients whose FOG did not respond to levodopa fell more frequently and had a significantly higher (worse) Hoehn and Yahr stage, significantly more impaired postural stability as assessed by the “pull subtest,” and the ability to stand on one leg. Locomotion as assessed by step length and the gait subtest were less impaired. We interpret this as implying that postural stability is compromised more than locomotion and that a deficiency in postural stability may be the principal problem in FOG.

The basic pattern of locomotion, the rhythmical movement of the legs, is generated in the spinal cord. Central pattern generators for locomotion are neural networks spread over several spinal segments [1, 18, 19]. A hierarchy of supraspinal centers sends descending commands to the spinal central pattern generators. This supraspinal command network is necessary for initiating gait, turning, stopping, and avoiding obstacles [1, 18, 19]. The spinal central pattern generators for locomotion plus the supraspinal command system constitute the* locomotor *network.

The* locomotor* network probably interacts with a* postural* stability network that includes position sensors in muscles and tendons, the otoliths and statoliths, the vestibular nuclei, the pedunculopontine nuclei (PPN), the basal ganglia, the thalami, and the frontal and parietal lobes [23–32]. The locomotor and balance networks include the limbic system, which may explain, in part, the association of FOG with anxiety [33, 34].

FOG in typical PD initially responds to levodopa but later may not [35–42]. This suggests that, as PD progresses, nondopaminergic (noradrenergic, cholinergic) and dopaminergic systems are disrupted.

The locomotor and postural stability networks include the PPN [43, 44], which are disrupted in typical PD and atypical parkinsonism, especially PSP [45]. The PPN, a mainly nondopaminergic nucleus, may be responsible, in part, for the impaired postural stability associated with FOG. Thus, infarction of the PPN results in FOG [46], and deep brain stimulation of the PPN may result in improvement in FOG [47].

Disruption of the PPN and other nondopaminergic nuclei (cholinergic, noradrenergic) mediating postural stability, may underlie FOG not responsive to levodopa.

## Figures and Tables

**Table 1:** Demographics of freezing of gait (FOG) patients unresponsive to levodopa compared to those of FOG patients responsive to levodopa.

Patient demographics	FOG patients unresponsive to levodopa (*N* = 18)^*^	FOG patients responsive to levodopa (*N* = 21)	Significance^†^

Age (yrs)	66.9 ± 7.1	67.4 ± 6.7	NS
Disease duration (yrs)	3.6 ± 0.9	4.2 ± 0.7	*P* = 0.036
Hoehn and Yahr stage ≥4	7/18	2/21	Odds ratio 6.05, 95% CI 1.06–34.4; *P* = **0.043**
≥2 falls/month	15/18	11/21	Odds ratio 4.6, 95% CI 1.01–20.5; *P* = **0.05**

^*^In the group, 12 patients had atypical parkinsonism and 6 had PD.

^†^Significant values are indicated in bold.

FOG: freezing of gait; PD: Parkinson's disease.

**Table 2:** Results of postural stability and locomotion tests on freezing of gait (FOG) patients unresponsive to levodopa compared to patients responsive to levodopa.

Tests	FOG patients unresponsive to levodopa (*N* = 18) Number Abnl Test/total number	FOG patients responsive to levodopa (*N* = 21) Number Abnl Test/total number	Significance^†^
FOG subtest >2	11/18	4/21	Odds ratio 6.7, 95% CI 1.58–28.3; *P* = **0.01**
Pull subtest ≥3	15/18	11/21	Odds ratio 4.6, 95% CI 1.00–20.5; *P* = **0.05**
Stand on one leg <3 seconds	15/18	11/21	Odds ratio 4.6, 95% CI 3.1–20.1; *P* = **0.05**
Gait subtest ≥2	10/18	6/21	Odds ratio 3.1; NS
Step length (meters)	0.45 ± 0.06	0.50 ± 0.404	*P* = **0.05 **

Abnl: abnormal; FOG: freezing of gait.

^†^Significant values are indicated in bold.

## References

[1] Nutt J. G., Bloem B. R., Giladi N., Hallett M., Horak F. B., Nieuwboer A. (2011). Freezing of gait: moving forward on a mysterious clinical phenomenon. *The Lancet Neurology*.

[2] Almeida Q. J., Lebold C. A. (2010). Freezing of gait in Parkinson's disease: a perceptual cause for a motor impairment?. *Journal of Neurology, Neurosurgery & Psychiatry*.

[3] Chee R., Murphy A., Danoudis M., Georgiou-Karistianis N., Iansek R. (2009). Gait freezing in Parkinson's disease and the stride length sequence effect interaction. *Brain*.

[4] Hausdorff J. M., Schaafsma J. D., Balash Y., Bartels A. L., Gurevich T., Giladi N. (2003). Impaired regulation of stride variability in Parkinson's disease subjects with freezing of gait. *Experimental Brain Research*.

[5] Giladi N., McDermott M. P., Fahn S. (2001). Freezing of gait in PD: prospective assessment in the DATATOP cohort. *Neurology*.

[6] Giladi N., McMahon D., Przedborski S. (1992). Motor blocks in Parkinson's disease. *Neurology*.

[7] Giladi N., Treves T. A., Simon E. S. (2001). Freezing of gait in patients with advanced Parkinson's disease. *Journal of Neural Transmission*.

[8] Lieberman A., Krishnamurthi N., Dhall R., Salins N., Pan D., Deep A. (2014). Comparison of Parkinson disease patients who fell once with patients who fell more than once (recurrent fallers). *Journal of Alzheimer's Disease & Parkinsonism*.

[9] Lieberman A. (2014). Falls in Parkinson disease: the relevance of short steps. *Journal of Novel Physiotherapies*.

[10] Voss T. S., Elm J. J., Wielinski C. L. (2012). Fall frequency and risk assessment in early Parkinson's disease. *Parkinsonism and Related Disorders*.

[11] Wood B. H., Bilclough J. A., Bowron A., Walker R. W. (2002). Incidence and prediction of falls in Parkinson's disease: a prospective multidisciplinary study. *Journal of Neurology, Neurosurgery and Psychiatry*.

[12] Gurevich T., Giladi N. (2003). Freezing of gait in multiple system atrophy (MSA). *Parkinsonism and Related Disorders*.

[13] Achiron A., Ziv I., Goren M. (1993). Primary progressive freezing gait. *Movement Disorders*.

[14] Factor S. A., Jennings D. L., Molho E. S., Marek K. L. (2002). The natural history of the syndrome of primary progressive freezing gait. *Archives of Neurology*.

[15] Hughes A. J., Daniel S. E., Ben-Shlomo Y., Lees A. J. (2002). The accuracy of diagnosis of parkinsonian syndromes in a specialist movement disorder service. *Brain*.

[16] Gelb D. J., Oliver E., Gilman S. (1999). Diagnostic criteria for Parkinson disease. *Archives of Neurology*.

[17] Abdo W. F., Borm G. F., Munneke M., Verbeek M. M., Esselink R. A. J., Bloem B. R. (2006). Ten steps to identify atypical parkinsonism. *Journal of Neurology, Neurosurgery and Psychiatry*.

[18] Müller J., Seppi K., Stefanova N., Poewe W., Litvan I., Wenning G. K. (2002). Freezing of gait in postmortem-confirmed atypical parkinsonism. *Movement Disorders*.

[19] Clarac F. (2008). Some historical reflections on the neural control of locomotion. *Brain Research Reviews*.

[20] Goetz C. G., Tilley B. C., Shaftman S. R. (2008). Movement Disorder Society-sponsored revision of the Unified Parkinson's Disease Rating Scale (MDS-UPDRS): scale presentation and clinimetric testing results. *Movement Disorders*.

[21] Lieberman A., Dhanani S., Dhall R., Pan D. (2012). A quick and easy-to-use clinical scale to assess balance in Parkinson's disease. *Journal of Parkinsonism & Restless Legs Syndrome*.

[22] Cheng F.-Y., Yang Y.-R., Wang C.-J. (2014). Factors influencing turning and its relationship with falls in individuals with Parkinson's disease. *PLoS ONE*.

[23] Peterson D. S., Pickett K. A., Duncan R., Perlmutter J., Earhart G. M. (2014). Gait-related brain activity in people with Parkinson disease with freezing of gait. *PLoS ONE*.

[24] Vandenbossche J., Deroost N., Soetens E. (2012). Freezing of gait in Parkinson's disease: disturbances in automaticity and control. *Frontiers in Human Neuroscience*.

[25] Plotnik M., Giladi N., Balash Y., Peretz C., Hausdorff J. M. (2005). Is freezing of gait in Parkinson's disease related to asymmetric motor function?. *Annals of Neurology*.

[26] Plotnik M., Giladi N., Hausdorff J. M. (2008). Bilateral coordination of walking and freezing of gait in Parkinson's disease. *European Journal of Neuroscience*.

[27] Shine J. M., Moustafa A. A., Matar E., Frank M. J., Lewis S. J. G. (2013). The role of frontostriatal impairment in freezing of gait in Parkinson's disease. *Frontiers in Systems Neuroscience*.

[28] Shine J. M., Matar E., Ward P. B. (2013). Freezing of gait in Parkinson's disease is associated with functional decoupling between the cognitive control network and the basal ganglia. *Brain*.

[29] Amboni M., Barone P., Iuppariello L. (2012). Gait patterns in Parkinsonian patients with or without mild cognitive impairment. *Movement Disorders*.

[30] Kostić V. S., Agosta F., Pievani M. (2012). Pattern of brain tissue loss associated with freezing of gait in Parkinson disease. *Neurology*.

[31] Herman T., Rosenberg-Katz K., Jacob Y., Giladi N., Hausdorff J. M. (2014). Gray matter atrophy and freezing of gait in Parkinson's disease: is the evidence black-on-white?. *Movement Disorders*.

[32] Muralidharan V., Balasubramani P. P., Chakravarthy V. S., Lewis S. J. G., Moustafa A. A. (2014). A computational model of altered gait patterns in parkinson's disease patients negotiating narrow doorways. *Frontiers in Computational Neuroscience*.

[33] Giladi N., Hausdorff J. M. (2006). The role of mental function in the pathogenesis of freezing of gait in Parkinson's disease. *Journal of the Neurological Sciences*.

[34] Lieberman A. (2006). Are Freezing of Gait (FOG) and panic related?. *Journal of the Neurological Sciences*.

[35] Bartels A. L., de Jong B. M., Giladi N. (2006). Striatal dopa and glucose metabolism in PD patients with freezing of gait. *Movement Disorders*.

[36] Schaafsma J. D., Balash Y., Gurevich T., Bartels A. L., Hausdorff J. M., Giladi N. (2003). Characterization of freezing of gait subtypes and the response of each to levodopa in Parkinson's disease. *European Journal of Neurology*.

[37] Espay A. J., Giuffrida J. P., Chen R. (2011). Differential response of speed, amplitude, and rhythm to dopaminergic medications in Parkinson's disease. *Movement Disorders*.

[38] Shahar T., Gadoth A., Nossek E., Giladi N., Ram Z., Maimon S. (2012). Reversible freezing of gait caused by dural arteriovenous fistula and congestion of the globus pallidus. *Movement Disorders*.

[39] Davis J. T., Lyons K. E., Pahwa R. (2006). Freezing of gait after bilateral subthalamic nucleus stimulation for Parkinson's disease. *Clinical Neurology and Neurosurgery*.

[40] Fasano A., Herzog J., Seifert E. (2011). Modulation of gait coordination by subthalamic stimulation improves freezing of gait. *Movement Disorders*.

[41] Moreau C., Defebvre L., Destée A. (2008). STN-DBS frequency effects on freezing of gait in advanced Parkinson disease. *Neurology*.

[42] Vercruysse S., Vandenberghe W., Münks L., Nuttin B., Devos H., Nieuwboer A. (2014). Effects of deep brain stimulation of the subthalamic nucleus on freezing of gait in Parkinson's disease: a prospective controlled study. *Journal of Neurology, Neurosurgery and Psychiatry*.

[43] Fraix V., Bastin J., David O. (2013). Pedunculopontine nucleus area oscillations during stance, stepping and freezing in Parkinson's disease. *PLoS ONE*.

[44] Schweder P. M., Hansen P. C., Green A. L., Quaghebeur G., Stein J., Aziz T. Z. (2010). Connectivity of the pedunculopontine nucleus in parkinsonian freezing of gait. *NeuroReport*.

[45] Warren N. M., Piggott M. A., Perry E. K., Burn D. J. (2005). Cholinergic systems in progressive supranuclear palsy. *Brain*.

[46] Ferraye M. U., Debû B., Fraix V. (2010). Effects of pedunculopontine nucleus area stimulation on gait disorders in Parkinson's disease. *Brain*.

[47] Kinno R., Yamazaki T., Owan Y., Uchiyama M., Kii Y., Fukui T. (2013). Freezing of gait caused by infarction in the unilateral genu of the corpus callosum. *Neurology and Clinical Neuroscience*.

